# Editorial: Surgical and non-surgical intervention of congenital heart disease management in developing and developed countries

**DOI:** 10.3389/fcvm.2025.1706239

**Published:** 2025-10-14

**Authors:** Brian Mendel, Inga Voges, Daniel De Wolf, Francisco Javier Ruperti-Repilado, Raymond N. Haddad, Edoardo Zancanaro

**Affiliations:** ^1^ Department of Cardiology and Vascular Medicine, National Cardiovascular Center Harapan Kita, Universitas Indonesia, Jakarta, Indonesia; ^2^ German Centre for Cardiovascular Research (DZHK), Kiel, Germany; ^3^ Department of Congenital Heart Disease and Pediatric Cardiology, University Hospital Schleswig-Holstein, Kiel, Germany; ^4^ Department of Pediatric Cardiology, University Hospitals Brussel and Gent, Ghent, Belgium; ^5^ Department of Cardiology, University Hospital Basel, University of Basel, Basel, Switzerland; ^6^ Cardiovascular Research Institute Basel, University Hospital Basel, Basel, Switzerland; ^7^ Filière des Cardiopathies Congénitales Enfant Adultes, Hôpital Marie Lannelongue, Centre Constitutif du Réseau Maladies Rares Malformations Cardiaques Congénitales Complexes-M3C, Hôpitaux Saint Joseph et Marie Lannelongue, Le Plessis Robinson, France; ^8^ Cardiovascular Research Institute, CARIM, University of Maastricht, Maastricht, Netherlands

**Keywords:** catheter-based intervention, developing country, late presenter, minimal-invasive, new device, prenatal intervention

**Editorial on the Research Topic**
Surgical and non-surgical intervention of congenital heart disease management in developing and developed countries

The management of congenital heart disease (CHD) has progressed substantially, with remarkable advances in transcatheter interventions and minimally invasive surgical techniques in recent decades. In this evolving landscape, continual adaptation to new devices and procedural innovations is essential.

Fluoroscopy inevitably exposes patients to ionizing radiation, carrying both stochastic and deterministic risks, which are of particular concern in small infants and pregnant women. Prakoso et al. analyzed 339 patients with median ASD diameter was 20 mm and the median device size deployed was 26 mm. Notably, 248 patients (73.1%) were categorized as having complex ASDs. The device implantation success rate was 98.9% in simple ASDs and 97.1% in complex ASDs These findings demonstrate that zero-fluoroscopyASD closure is both feasible and effective for simple and complex defects.

Hypoplastic left heart syndrome (HLHS) represents one of the most severe forms of CHD. Burleigh et al. retrospectively analyzed consecutive cardiovascular magnetic resonance (CMR) examinations in 80 patients (22 females), each undergoing two (*n* = 80) or three (*n* = 45) serial studies. Across all examinations, median regurgitant fraction (RF) of the neo-aortic valve remained mild (RF <20%), with no significant progression over time. These findings demonstrate that neo-aortic valve function is generally preserved in HLHS patients following TCPC.

Liu et al. included 70 VSD patients with 35 of them had a right vertical axillary incision and 35 had, median sternotomy. No significant differences were observed between the two groups for age or weight. Compared with the median sternotomy group, the right vertical axillary incision group demonstrated a significantly shorter incision length and lower median postoperative drainage. These findings indicate that VSD repair via a right vertical axillary incision is a safe alternative to median sternotomy with the additional advantage of reduced surgical trauma and improved cosmetic outcomes.

Khalek et al. analysed seven patients with cor triatriatum. All patients had cor triatriatum sinister, most frequently presenting with respiratory symptoms. Surgical membrane resection was performed in all operated patients, with postoperative outcomes showing significant symptomatic improvement.

The intricate anatomy of doubly committed subarterial VSD, coupled with their proximity to valvular and conduction tissues, as well as concerns regarding radiation and contrast exposure, renders transcatheter closure particularly challenging in pediatric patients. Prakoso et al. reported the case of a symptomatic 18-month-old boy. Transthoracic echocardiography demonstrated a 4–5 mm left-to-right shunting subarterial VSD. Retrograde deployment of a Konar-MF VSD occluder (7/5 mm) was successfully achieved, resulting in complete closure of the VSD.

Despite the improvements, adults with CHD (ACHD) remain burdened by high morbidity and mortality, frequent hospital readmissions, and substantial healthcare costs. In the study by Bieze et al., 1,381 ACHD patients underwent cardiac surgery, of whom 292 (20.5%) were >50 years. Among older patients, increased complication risk was associated with longer CPB time and higher preoperative creatinine levels. One-year mortality did not differ significantly between groups. These findings suggest that with appropriate patient selection and preoperative optimization, surgical outcomes in aging ACHD patients remain favorable, with overall operative risks kept at acceptable levels.

Siagian and Christianto et al. examined 501 patients who underwent ToF repair. They reported low reoperation (6.5%) and 30-day mortality rates (4.7%); however, prolonged length of stay (92.2%) and a high overall complication rate (84%) were observed. Among the indices evaluated, preoperative NLR demonstrated the greatest predictive value for postoperative complications, despite limited sensitivity and specificity. Given its affordability and wide availability, NLR may represent a practical tool for preoperative risk stratification and postoperative surveillance in ToF patients.

David et al. reported the case of a two-month-old infant, who was referred for dermatological evaluation of a growing hemifacial hemangioma. Transthoracic echocardiography revealed severe coarctation of the aorta and aortic arch hypoplasia. Intraoperative findings of an extended coarcted segment and extensive collateral circulation precluded this approach. Resection of the stenotic segment with end-to-end anastomosis and interposition of a Dacron patch was performed. The patient recovered well, and the postoperative results were favorable.

Jing et al. analyzed 783 patients, of whom 434 underwent median sternotomy (MS) and 349 underwent of right subaxillary small incision (RSSI). After propensity score matching, 282 patients were included in each group. No significant differences were observed between the groups in terms of residual ASDs or VSDs, peak airway pressure, PaO_2_/FiO_2_ ratio, or PaCO_2_ levels prior to ICU transfer and extubation. However, the RSSI group demonstrated significantly shorter durations of mechanical ventilation, ICU stay, and overall hospitalization. Perioperative complication rates were comparable between the two groups.

The Amplatzer Membranous VSD Occluder was withdrawn from clinical use following reports of complete heart block in ∼5%–10% of cases. In a multicenter study by Elafifi et al. using newly approved soft-profile device, Konar MFO; they showed percutaneous VSD closure using the MFO demonstrated high procedural success, acceptable complication rates, and favorable short- to mid-term outcomes, confirming its safety, efficacy, and feasibility.

Cardiopulmonary bypass during open-heart surgery elicits a robust systemic inflammatory response triggered by several mechanisms. In. a study by Chaiwiriyawong et al. looking at 127 patients (median age, 44.4 months), 37 (29.1%) had a Risk Adjustment for Congenital Heart Surgery (RACHS) score ≥3, and 26 (20.4%) experienced low cardiac output syndrome (LCOS)-related outcomes. However, VACO_2_ was not significantly associated with LCOS-related outcomes in pediatric patients undergoing CPB. Sustained elevation of VACO₂ early after surgery correlated with prolonged inotrope use and extended ICU stay.

A recent status report on CHD in India underscores its prominence as the most common congenital anomaly. Tandon et al. study showed in 422 pediatric patients who were admitted, 386 underwent cardiac surgery. Among the 386 operated patients, 16 (4.1%) died. The most common surgical procedures performed included VSD closure, patent ductus arteriosus ligation, and TOF repair.

Huang et al. studied a cohort of 210 patients, in which 84.29% presented with mild pulmonary arterial hypertension (PAH), 8.57% with moderate, and 7.14% with severe PAH. Device implantation was successful in 98.10% of cases. Early adverse events occurred in 12.14% (*n* = 25), with residual shunt and arrhythmia being the most frequent complications (2.91% each, *n* = 6). Most complications were transient and clinically insignificant, except for two cases of residual shunt and one case of complete left bundle branch block necessitating device removal.

Infants with complex CHDs often require multiple staged palliative and corrective surgeries early in life, with prolonged hospitalizations in the cardiac intensive care unit (CICU). Xia et al. showed that children from Cluster 1 (Active and Collaborative) had the highest quality of life, whereas those in Cluster 2 (Chaotic and Nervous) had the lowest (73.93 ± 12.71 vs. 59.03 ± 18.70). Unplanned readmission rates were significantly greater in Clusters 2 and 4 (18.52% and 22.11%) compared with Clusters 1 and 3 (4.05% and 3.57%). Family management style significantly influenced postoperative outcomes.

